# Claudin-4 Overexpression Predicts Poor Survival and Platinum Resistance in Epithelial Ovarian Cancer: A Potential Biomarker for Clinical Decision-Making †

**DOI:** 10.3390/diagnostics15243163

**Published:** 2025-12-11

**Authors:** Özlem Kutlu, Damla Günenç, Duygu Ayaz, Özlem Özdemir, Halil Taşkaynatan, Celal Akdemir, Muzaffer Sancı

**Affiliations:** 1 Department of Medical Oncology, Izmir City Hospital, Izmir 35540, Turkey; ozdemirozlem.md@gmail.com (Ö.Ö.); haliltaskaynatan@gmail.com (H.T.); 2 Department of Medical Oncology, Hatay Training and Research Hospital, Hatay 31060, Turkey; d.gunenc@yahoo.com.tr; 3 Department of Pathology, Izmir Tepecik Training and Research Hospital, Izmir 35180, Turkey; ayazduygu@yahoo.com; 4 Department of Gynecologic Oncology Surgery, Izmir City Hospital, Izmir 35540, Turkey; akdemircelal@gmail.com (C.A.); drsanci@yahoo.com (M.S.)

**Keywords:** epithelial ovarian cancer, claudin-4, platinum resistance, prognostic biomarker

## Abstract

**Background/Objectives**: Epithelial ovarian cancer (EOC) is a leading cause of death among forms of gynecologic cancer. Significant causes of mortality include high recurrence rates and the development of resistance to platinum-based chemotherapy. This highlights the need for reliable prognostic biomarkers to improve patient stratification and inform treatment decisions. Claudin-4, a tight junction protein frequently overexpressed in epithelial tumors, has been associated with tumor progression and resistance to chemotherapy. **Methods**: We retrospectively analyzed 83 patients with EOC who underwent debulking surgery. Claudin-4 expression was assessed by immunohistochemistry and categorized as high or low based on a semi-quantitative scoring system. Survival outcomes were evaluated using Kaplan–Meier analysis and Cox regression. Predictors of platinum resistance were examined using logistic regression. **Results**: High Claudin-4 expression was observed in 55.4% of cases and was associated with significantly shorter disease-free survival (DFS) (23 vs. 66 months, *p* = 0.00024) and overall survival (OS) (85 months vs. NR, *p* = 0.0031). In multivariable analysis, platinum resistance (DFS; HR 4.99, OS; HR 4.27) and high Claudin-4 expression (DFS; HR 2.46, OS; HR 3.59) were independent predictors of poor outcomes. Logistic regression further demonstrated that high Claudin-4 expression and interval debulking surgery were independent predictors of platinum resistance. **Conclusions**: High Claudin-4 expression was associated with inferior survival and an increased risk of platinum resistance in EOC. Our findings suggest that Claudin-4 may serve as a negative prognostic biomarker and a potential therapeutic target. Future prospective studies are warranted to further elucidate the underlying mechanisms and validate Claudin-4’s clinical utility.

## 1. Introduction

Ovarian cancer ranks as the seventh most common malignancy among women worldwide, with high mortality rates [1]. Approximately 90% of malignant ovarian tumors originate from epithelial cells [2,3,4]. The standard management of epithelial ovarian cancer (EOC) includes optimal cytoreductive surgery combined with platinum-based systemic chemotherapy [5,6]. While initial response rates exceed 80%, most patients eventually relapse, and a significant proportion develop resistance to platinum-based therapy [7]. Platinum resistance represents a major therapeutic challenge and is associated with poor survival outcomes [8]. Despite advances in therapeutic approaches such as anti-angiogenic agents and antibody–drug conjugates (ADCs), overall survival (OS) benefits remain modest, highlighting the urgent need for applicable biomarkers [8,9,10,11,12].

Recent therapeutic progress in EOC increasingly emphasizes biomarker-driven approaches to overcome platinum resistance. The clinical success of mirvetuximab soravtansine, an ADC targeting folate receptor alpha (FRα), exemplifies how molecular stratification can reshape treatment paradigms [10,11]. These developments highlight the importance of identifying molecular determinants that underlie chemoresistance and may serve as both prognostic and predictive biomarkers.

Among such molecular candidates, the Claudin family of tight junction proteins has attracted attention for its dual role in maintaining epithelial barrier integrity and modulating tumor biology [13]. Claudin-4 has emerged as one of the most clinically relevant members, showing paradoxical overexpression in several epithelial malignancies, including ovarian, breast, gastric, pancreatic, and hepatic cancers [14,15,16,17]. Beyond its structural function, Claudin-4 contributes to tumor invasion, motility, angiogenesis, and resistance to apoptosis [18,19,20,21]. In ovarian cancer, Claudin-4 is frequently mislocalized outside tight junctions, where it participates in aberrant signaling pathways that promote tumor progression and metastasis [22,23]. Increasing evidence suggests that elevated Claudin-4 expression is associated with worse prognosis in EOC and may modulate cellular responses to taxanes and platinum-based agents [24,25,26,27]. Moreover, Claudin-4 represents a promising therapeutic target beyond its role as a biomarker. Antibodies against Claudin-4 or related isoforms such as CLDN18.2 have demonstrated encouraging antitumor effects in preclinical and early-phase clinical studies [28]. Given its surface accessibility and involvement in DNA repair pathways, Claudin-4 may constitute a targetable molecular vulnerability in EOC biology [29]. However, clinical studies have reported inconsistent findings regarding its predictive and prognostic utility, highlighting the need for further investigation in well-defined patient cohorts.

To address this knowledge gap, we evaluated Claudin-4 expression in a uniformly treated, real-world EOC cohort receiving standard platinum-based chemotherapy. By correlating expression levels with platinum resistance and survival outcomes, we aimed to clarify its clinical relevance from both prognostic and predictive perspectives. Our findings may help refine patient stratification and support the development of biomarker-guided treatment strategies in EOC.

## 2. Materials and Methods

### 2.1. Study Design and Pat3ient Selection

This retrospective study was conducted at İzmir Tepecik Training and Research Hospital with the approval of Izmir City Hospital Ethics Committee (Approval No: 2024/73). Patients diagnosed with EOC who underwent debulking surgery between January 2017 and January 2024 were eligible for inclusion. Patients with low-grade serous histology, second primary malignancy, or insufficient follow-up data were excluded. Clinical data, including age at diagnosis, FIGO stage, histological subtype, residual disease status, germline BRCA mutation status, and treatment outcomes, were extracted from electronic medical records.

### 2.2. Definitions and Endpoints

Platinum resistance was defined as recurrence occurring within six months of completing first-line platinum-based chemotherapy. Residual tumor burden was categorized as optimal (R0; <1 cm) or suboptimal (R1; ≥1 cm) based on the maximal diameter of residual disease after surgery.

The primary endpoints included DFS and OS stratified by Claudin-4 expression levels, both measured from the time of diagnosis. The secondary endpoint was identification of predictive factors for platinum resistance.

### 2.3. Sample Preparation and Immunohistochemistry

Distal tubules and collecting duct sections of normal kidney tissue were utilized as positive controls. Negative controls consisted of parallel sections processed without the primary antibody (no-primary control). Fallopian tube tissue was used as the substrate for the negative control slide. Paraffin-embedded tumor tissues were sectioned at 5 µm thickness using a microtome. Slides were incubated at 60 °C for 15 min to soften the paraffin, followed by automated deparaffinization and rehydration using the xylene-free Ventana EZ Prep solution. Sections were treated with 0.3% hydrogen peroxide (H_2_O_2_) to block endogenous peroxidase activity. All immunohistochemical procedures were carried out on a fully automated BenchMark ULTRA instrument (Ventana Medical Systems, Inc., Tucson, AZ, USA). Heat-induced epitope retrieval (HIER) was performed at 98 °C in a Tris-based buffer (Cell Conditioning 1, pH 9) as per the manufacturer’s protocol. Slides were incubated for 30 min at 36 °C with a mouse monoclonal anti-Claudin-4 antibody (clone ER417, 1:150 dilution; Epitomics, Fremont, CA, USA). Detection of antigen–antibody complexes was achieved using the OptiView DAB IHC Detection Kit (Ventana Medical Systems, Inc.). Subsequently, sections were counterstained with hematoxylin and coverslipped using Entellan^®^ mounting medium (Merck, Darmstadt, Germany) on a Dako CoverStainer (Agilent Technologies, Santa Clara, CA, USA). All staining and mounting steps were performed at room temperature, protected from dust and direct light. Stained slides were then air-dried horizontally for 24 h prior to microscopic evaluation.

Immunohistochemical scoring of Claudin-4 was performed in an exploratory manner, given the absence of a standardized system in EOC. A semi-quantitative method, previously applied in other epithelial malignancies, was adopted [30]. Initially, staining intensity, scored from 0 to 3, was evaluated at 10× magnification: 0 = negative, 1 = weak, 2 = moderate, and 3 = strong. Staining extent was quantified according to the percentage of positively cells stained at 40× magnification: 1 = <25%, 2 = 25–50%, 3 = 51–75%, 4 = >75%. For percentage scoring, both membranous and cytoplasmic staining patterns were jointly evaluated.

**Figure 1:**
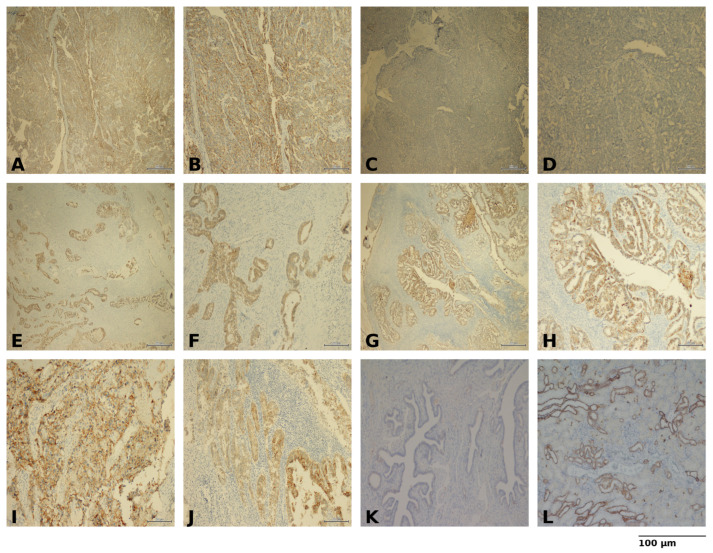
Representative immunohistochemical staining of epithelial ovarian cancer (**A**) High Claudin-4 expression, ×40 (**B**) High Claudin-4 expression, ×100 (**C**) Low Claudin-4 expression, ×40 (**D**) Low Claudin-4 expression, ×100 (**E**) Cytoplasmic Claudin-4 staining, ×40 (**F**) Cytoplasmic Claudin-4 staining, ×100 (**G**) Predominantly membranous Claudin-4 staining, ×40 (**H**) Predominantly membranous Claudin-4 staining, ×100 (**I**) Clear cell carcinoma morphology with Claudin-4 staining, ×100 (**J**) Endometrioid carcinoma morphology with Claudin-4 staining, ×100 (**K**) Negative control (no primary antibody), ×100 (**L**) Positive control, ×100. Scale bars = 100 µm.

For each case, a total of 50 high-power fields (HPFs) at 400× magnification were examined, and the entire tumor area was systematically scanned to avoid field-selection bias. Areas showing necrosis, crush artifacts, or suboptimal fixation were excluded from analysis. The final score was calculated by summing intensity and extent. Scores of 0–3 were classified as low expression, and 4–7 as high expression (Figure 1). All slides were independently reviewed by a gynecologic pathologist blinded to clinical outcomes. Additionally, all immunohistochemical evaluations were performed by a single gynecologic pathologist with more than 10 years of experience in gynecologic oncology pathology.

### 2.4. Statistical Analysis

Descriptive statistics were presented as numbers and percentages for categorical variables and as mean ± standard deviation (SD) or median (interquartile range, IQR) for continuous variables. Chi-square test or Fisher’s exact test was used for intergroup comparisons of categorical data. DFS and OS were estimated using the Kaplan–Meier method, and survival differences between groups were assessed with the log-rank test. Factors associated with survival were evaluated using Cox proportional hazards regression models. Predictors of platinum resistance were analyzed with logistic regression. Statistical analyses were performed using Jamovi (version 2.7.2.0) and GraphPad Prism (version 10.5.0). A two-sided *p*-value < 0.05 was considered statistically significant.

## 3. Results

### 3.1. Patient Characteristics

A total of 83 patients with EOC were included in the analysis. The median age at diagnosis was 55 years (IQR: 45–61). The majority of patients (86.7%) had serous histology, and 67.5% were diagnosed at FIGO stage III–IV. All patients underwent debulking surgery, either as primary (66.3%) or interval (33.7%) procedures, with optimal debulking (R0) achieved in 81.9% of cases. Germline BRCA1/2 mutation status was available for 47 patients (56.7%), and 15 of them (18.1% of the cohort) tested positive. Patients completed at least six cycles of platinum-based chemotherapy, except for one who progressed during neoadjuvant treatment and another who received only three adjuvant cycles for stage IA disease.

Patients were categorized into two groups based on their Claudin-4 expression status: High-Claudin-4 (*N* = 46, 55.4%) and Low-Claudin-4 (*N* = 37, 44.6%). Baseline clinicopathologic characteristics, including age, stage, histology, BRCA mutation, surgical timing, and residual disease, were similar between groups (*p* > 0.05 for all; Table 1).

### 3.2. Survival Outcomes

More than half of the study population (57.8%) experienced recurrence, and 22.9% died during a median of 53 months (95% CI: 49–61) follow-up period. The median DFS for the overall cohort was 36 months (95% CI: 30–63), while the median OS was not reached (95% CI: 85–NR).

**Figure 2:**
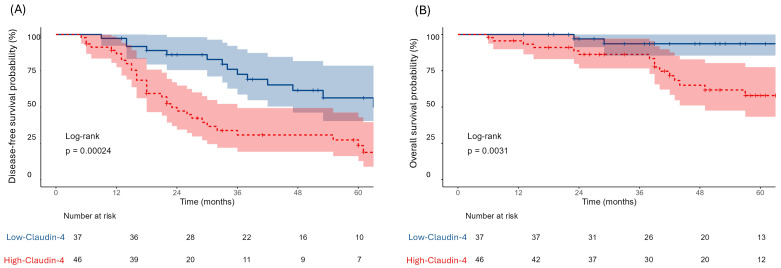
Kaplan–Meier curves demonstrating DFS (**A**) and OS (**B**) stratified by Claudin-4 expression. Blue lines represent the Low-Claudin-4 group and red lines represent the High-Claudin-4 group.

Recurrence and mortality were more prevalent in High-Claudin-4 group (*p* = 0.004 for both; Table 1). Claudin-4 expression also strongly influenced survival outcomes. Patients in Low-Claudin-4 group exhibited more favorable outcomes, with a median DFS of 66 months (95% CI: 47–NR) compared to 23 months (95% CI: 18–36) High-Claudin-4 group (*p* = 0.00024). Similarly, median OS was not reached in Low-Claudin-4 group, whereas it was 85 months (95% CI: 49–NR) for High-Claudin-4 group (*p* = 0.0031, Figure 2).

In univariate Cox regression (Table 2), advanced stage, interval debulking surgery, platinum resistance, and high Claudin-4 expression were significantly associated with inferior DFS and OS. Among these, platinum resistance emerged as the strongest prognostic factor for both DFS (HR 11.88, 95% CI 5.17–26.53, *p* < 0.001) and OS (HR 8.89, 95% CI 3.17–23.63, *p* < 0.001). High Claudin-4 expression was also highly correlated with poor DFS (HR 3.03, 95% CI: 1.63–5.64, *p* < 0.001) and OS (HR 5.47, 95% CI: 1.78–23.89, *p* = 0.002). Although a trend toward improved DFS was observed in patients with non-serous histology (HR 0.25, 95% CI 0.04–1.04, *p* = 0.056), this did not reach statistical significance. Age, residual disease status, and BRCA mutation were not associated with outcomes and excluded from multivariate analysis.

In the multivariate model (Table 3), platinum resistance remained the most powerful independent prognostic factor of DFS (HR 4.99, 95% CI 2.06–12.0, *p* = 0.0005) and OS (HR 4.27, 95% CI 1.36–13.53, *p* = 0.014). High Claudin-4 expression also retained significance for both DFS (HR 2.46, 95% CI 1.25–4.98, *p* = 0.009) and OS (HR 3.59, 95% CI 1.02–17.26, *p* = 0.046). Interval surgery independently predicted worse DFS (HR 2.07, 95% CI 1.02–4.25, *p* = 0.043), but not OS. Disease stage lost statistical significance in multivariate analysis.

### 3.3. Platinum Resistance

Platinum resistance occurred in 11 (13.3%) patients and was more frequent in High-Claudin-4 group (*p* = 0.019). Median age was comparable between platinum-resistant (56 years, IQR: 43–61) and platinum-sensitive patients (54 years, IQR: 46.25–62.5).

In univariate logistic regression (Table 4), high Claudin-4 expression (OR 10.0, 95% CI: 1.78–188.3, *p* = 0.006) and interval debulking surgery (OR 4.25, 95% CI: 1.16–17.7, *p* = 0.029) were significantly associated with platinum resistance. In the multivariate model (Table 4), both Claudin-4 expression (OR 9.88, 95% CI: 1.70–188.8, *p* = 0.008) and interval surgery (OR 4.19, 95% CI: 1.08–18.4, *p* = 0.039) remained independent predictors. The model showed good calibration (Hosmer–Lemeshow *p* = 0.98) and discriminative ability (AUC 0.79, 95% CI: 0.67–0.91, *p* = 0.002), with modest explanatory power (pseudo R^2^ = 0.14).

## 4. Discussion

In this study, we investigated the clinical relevance of Claudin-4 expression in EOC, focusing on its association with platinum resistance and survival outcomes. Our findings demonstrated that high Claudin-4 expression was significantly associated with shorter DFS and OS, supporting its role as a negative prognostic biomarker. Furthermore, Claudin-4 overexpression, along with interval debulking surgery, independently predicted platinum resistance, while platinum resistance itself emerged as the strongest determinant of poor survival.

Given that platinum resistance is a key determinant of prognosis in EOC, the poorer outcomes observed in Claudin-4–high patients may reflect underlying chemoresistant biology. Breed et al. showed that apoptosis was enhanced in Claudin-4–positive ovarian cancer cells when paclitaxel was combined with a Claudin-4–disrupting peptide, but not with cisplatin, suggesting drug-specific resistance mechanisms [25]. Casagrande et al. demonstrated that CD44^+^ tumor cells expressed higher levels of Claudin-4 and this subpopulation showed greater resistance to carboplatin and paclitaxel [31]. Similarly, Gao et al. demonstrated that inhibition of Claudin-3 and -4 enhanced tumor cell sensitivity to platinum and taxanes, indicating a functional contribution to chemoresistance [32]. Complementary proteomic and transcriptomic analyses have identified Claudin-4 as one of the most upregulated proteins in cisplatin-resistant ovarian cancer cells, linking its overexpression to impaired DNA-damage response, epithelial–mesenchymal transition signatures, and restored cisplatin sensitivity upon silencing [24,33,34]. Collectively, these data suggest that Claudin-4 contributes functionally to drug resistance and may act as a resistance-associated marker [35].

Histopathologic studies have reinforced the malignant specificity of Claudin-4, providing a biological background for its potential clinical utility. Rangel et al. showed that Claudin-3 and Claudin-4 were frequently overexpressed in ovarian carcinomas but not in benign cystadenomas [22]. Similarly, Hough et al. identified Claudin-4 among differentially expressed genes in ovarian cancer compared to normal epithelium [17].

Despite these strong experimental findings, clinical data remain conflicting. Litkouh et al. found no association between Claudin-4 expression and platinum sensitivity or survival in high-grade serous ovarian cancers [36]. Similarly, in a cohort of 140 EOC patients, no significant correlation was reported with platinum resistance, although low Claudin-4 expression was associated with improved DFS and OS [37]. Another study using exploratory IHC scoring in a mixed-grade ovarian carcinoma cohort reported prognostic but not predictive associations [38]. Such discrepancies may reflect methodological differences, heterogeneous patient cohorts, or lack of standardized scoring systems for Claudin-4. Moreover, definitions of platinum resistance vary across studies, which may further contribute to inconsistent results.

Beyond its prognostic implications, Claudin-4 has also been investigated as a potential therapeutic target and a modulator of DNA repair pathways in EOC [39]. Monoclonal antibodies, such as KM3900 and 4D3, directed against the extracellular domains of Claudin-4, have demonstrated antitumor effects in preclinical models by disrupting tight junctions, enhancing drug delivery, and activating immune responses [18,40,41,42]. Yamamoto et al. demonstrated that loss of Claudin-4 impaired the DNA repair mechanisms and antiproliferative effects of PARP inhibitors in both in vitro and ex vivo ovarian cancer models. In contrast, tumors expressing high levels of Claudin-4 showed reduced response to PARP inhibitors [33].

Our findings provide evidence supporting the use of Claudin-4 as a negative prognostic marker with potential clinical utility in EOC. Claudin-4 immunohistochemistry could serve as a cost-effective biomarker to stratify patients at diagnosis, potentially guiding treatment decisions. Furthermore, given its cell surface localization and functional role in tumor biology, Claudin-4 represents an attractive therapeutic target, as evidenced by ongoing clinical trials and early translational studies investigating Claudin-targeted antibodies and ADCs in gastrointestinal and gynecologic cancers [43].

Our study further demonstrated that interval debulking surgery emerged as an independent predictor of platinum resistance. This observation supports prior evidence suggesting that delayed cytoreductive procedures may promote the development of chemoresistant tumor clones, underscoring the importance of surgical timing in shaping tumor biology [44].

Several limitations of our study should be acknowledged. Its retrospective, single-center design and modest sample size limit generalizability. Wide confidence intervals observed for some estimates further reflect a potential influence of outliers. Although the multivariate model demonstrated reasonable discrimination, the modest explanatory power anticipates the potential contribution of additional factors. Finally, we were unable to perform longitudinal monitoring of Claudin-4 expression, and Claudin-4 scoring was exploratory, as no standardized cut-off exists for EOC.

Despite certain limitations, our study also has notable strengths. First, it provides real-world data on Claudin-4 expression and clinical outcomes in EOC, complementing previous experimental observations. Second, unlike most preclinical studies focused on single agents, our analysis evaluated resistance under standard combination chemotherapy, thereby offering more clinically relevant insights. Unlike prior investigations largely limited to bioinformatic or small-scale preclinical approaches, our study provides clinical validation of Claudin-4 expression in a uniformly treated, real-world EOC cohort, demonstrating its dual prognostic and predictive relevance. This integrated approach bridges experimental findings with clinical applicability and underscores the translational importance of Claudin-4 in ovarian cancer. Future prospective, multicenter studies integrating Claudin-4 expression with genomic and transcriptomic profiling could further refine its prognostic and predictive potential in epithelial ovarian cancer.

## 5. Conclusions

Our results showed that high Claudin-4 expression is associated with poor survival and increased risk of platinum resistance in EOC. Overall, our hypothesis-generating study findings, combined with recent exploratory data [45], underscore the broader clinical relevance of Claudin-4 in EOC and justify the need for further research and external validation through larger, prospective cohorts.

## Figures and Tables

**Table 1:** Comparison of Clinicopathological Characteristics According to Claudin-4 Expression Levels.

Variables	Total (*N* = 83)	Low Claudin-4 (*N* = 37)	High Claudin-4 (*N* = 46)	*p*-Value ^a^
**Age (years)**				
Mean ± SD	54.24 ± 10.30	52.75 ± 8.58	55.43 ± 11.45	0.242 ^b^
<60	58 (69.9%)	29 (78.4%)	29 (63.0%)	0.130
≥60	25 (30.1%)	8 (21.6%)	17 (37.0%)	
**Histology**				
Serous	72 (86.7%)	33 (89.2%)	39 (84.8%)	0.747
Others *	11 (13.3%)	4 (10.8%)	7 (15.2%)	
**Stage (FIGO)**				
I–II	27 (32.5%)	15 (40.5%)	12 (26.1%)	0.162
III–IV	56 (67.5%)	22 (59.5%)	34 (73.9%)	
**gBRCA mutation ^†^**				
Negative	32 (38.6%)	13 (35.1%)	19 (41.3%)	0.696
Positive	15 (18.1%)	7 (18.9%)	8 (17.4%)	
**Timing of Surgery**				
Primary	55 (66.3%)	26 (70.3%)	29 (63.0%)	0.489
Interval	28 (33.7%)	11 (29.7%)	17 (37.0%)	
**Residual Disease**				
R0	68 (81.9%)	31 (83.8%)	37 (80.4%)	0.693
R1	15 (18.1%)	6 (16.2%)	9 (19.6%)	
**Platinum Resistance ^‡^**				
No	72 (86.7%)	36 (97.3%)	36 (78.3%)	**0.019**
Yes	11 (13.3%)	1 (2.7%)	10 (21.7%)	
**Recurrence**				
No	35 (42.2%)	22 (59.5%)	13 (28.3%)	**0.004**
Yes	48 (57.8%)	15 (40.5%)	33 (71.7%)	
**Mortality**				
No	64 (77.1%)	34 (91.9%)	30 (65.2%)	**0.004**
Yes	19 (22.9%)	3 (8.1%)	16 (34.8%)	

* Others included, clear cell carcinoma (*N* = 7) and endometrioid carcinoma (*N* = 4). ^†^ gBRCA mutation status evaluated for *N* = 47 patients. **^‡^** Platinum resistance is defined as resistance to first line platinum-based chemotherapy. Data are presented as *n* (%) for categorical variables and mean (±SD) for continuous variables. *p*-values calculated using ^a^ Chi-square or Fisher’s exact test and ^b^ Independent samples *t*-test. Bold headings are used for variable and group labels for clarity. Bold values indicate statistically significant differences at a level of ≤0.05. FIGO: International Federation of Gynecology and Obstetrics; gBRCA: germline BRCA.

**Table 2:** Univariate Cox proportional hazards regression for DFS and OS.

	Univariate					
Variables		*N*	DFS HR (95% CI)	*p*-Value	OS HR (95% CI)	*p*-Value
**Age (years)**	<60	58	Reference	—	Reference	—
	≥60	25	1.55 (0.86–2.76)	0.151	1.02 (0.36–2.70)	0.961
**Histology**	Serous	72	Reference	—	Reference	—
	Others *	11	0.25 (0.04–1.04)	0.056	0.45 (0.02–2.18)	0.377
**Stage (FIGO)**	I–II	27	Reference	—	Reference	—
	III–IV	56	4.30 (1.90–9.76)	**<0.001**	4.08 (1.17–25.75)	**0.025**
**gBRCA mutation ^†^**	Negative	32	Reference	—	Reference	—
	Positive	15	0.51 (0.21–1.14)	0.103	0.46 (0.02–5.10)	0.526
**Timing of Surgery**	Primary	55	Reference	—	Reference	—
	Interval	28	3.38 (1.91–6.00)	**<0.001**	2.86 (1.15–7.42)	**0.024**
**Platinum Resistance ^‡^**	No	72	Reference	—	Reference	—
	Yes	11	11.88 (5.17–26.53)	**<0.001**	8.89 (3.17–23.63)	**<0.001**
**Claudin-4 Expression**	Low	37	Reference	—	Reference	—
	High	46	3.03 (1.63–5.64)	**<0.001**	5.47 (1.78–23.89)	**0.002**
**Residual Disease**	R0	68	Reference	—	Reference	
	R1	15	1.38 (0.68–2.77)	0.369	0.56 (0.09–1.96)	0.405

* Others included, clear cell carcinoma (*N* = 7) and endometrioid carcinoma (*N* = 4). ^†^ gBRCA mutation status evaluated for *N* = 47 patients. ^‡^ Platinum resistance defined as resistance to first-line platinum-based chemotherapy. Data are presented as hazard ratios (HR) and 95% confidence intervals (95%CI). Bold headings are used for variable and group labels for clarity. Bold values indicate statistically significant differences at a statistical significance level of ≤0.05. FIGO: International Federation of Gynecology and Obstetrics; gBRCA: germline BRCA.

**Table 3:** Multivariate Cox proportional hazards regression for DFS and OS.

Variables		*N*	Multivariate			
			DFS		OS	
			HR (95% CI)	*p*-Value	HR (95% CI)	*p*-Value
**Stage (FIGO)**	I–II	27	Reference	—	Reference	—
	III–IV	56	2.25 (0.93–6.04)	0.072	1.81 (0.4–12.72)	0.464
**Timing of Surgery**	Primary	55	Reference	—	Reference	—
	Interval	28	2.07 (1.02–4.25)	**0.043**	1.53 (0.50–5.0)	0.456
**Platinum resistance ^‡^**	No	72	Reference	—	Reference	—
	Yes	11	4.99 (2.06–12.0)	**0.0005**	4.27 (1.36–13.53)	**0.014**
**Claudin-4 Expression**	Low	37	Reference	—	Reference	—
	High	46	2.46 (1.25–4.98)	**0.009**	3.59 (1.02–17.26)	**0.046**

^‡^ Platinum resistance defined as resistance to first-line platinum-based chemotherapy. Data are presented as hazard ratios (HR) and 95% confidence intervals (95%CI). Bold headings are used for variable and group labels for clarity. Bold values indicate statistically significant differences at a level of ≤0.05. FIGO: International Federation of Gynecology and Obstetrics.

**Table 4:** Logistic regression analysis of factors associated with platinum resistance.

Variables		Univariate OR (95% CI)	*p*-Value	Multivariate OR (95% CI)	*p*-Value
**Age (years)**	<60	Reference	—	—	—
	≥60	0.85 (0.17–3.27)	0.824	—	—
**Histology**	Serous	Reference	—	—	—
	Others *	0.62 (0.03–3.81)	0.648	—	—
**Stage (FIGO)**	I–II	Reference	—	—	—
	III–IV	2.39 (0.56–16.5)	0.254	—	—
**Timing of Surgery**	Primary	Reference	—	Reference	—
	Interval	4.25 (1.16–17.7)	**0.029**	4.19 (1.08–18.4)	**0.038**
**Residual Disease**	R0	Reference	—	—	—
	R1	1.01 (0.14–4.52)	0.992	—	—
**Claudin-4 Expression**	Low	Reference	—	Reference	—
	High	10.0 (1.78–188.3)	**0.006**	9.88 (1.70–188.8)	**0.008**

* Others included, clear cell carcinoma (*N* = 7) and endometrioid carcinoma (*N* = 4). Data are presented as odds ratios (OR) and 95% confidence intervals (95% CI). FIGO: International Federation of Gynecology and Obstetrics. Bold headings are used for variable and group labels for clarity. Bold numerical values indicate statistically significant results (*p* < 0.05).

## Data Availability

The data presented in this study are available on reasonable request from the corresponding author. The data are not publicly available due to privacy and ethical restrictions.

## Abbreviations

The following abbreviations are used in this manuscript:

EOC	Epithelial ovarian cancer
ADCs	Antibody–drug conjugates
FRα	Folate receptor alpha
OS	Overall survival
DFS	Disease-free survival
SD	Standard deviation
IQR	Interquartile range
HR	Hazard ratio
CI	Confidence intervals
OR	Odds ratio
FIGO	International Federation of Gynecology and Obstetrics
